# Authors’ response

**DOI:** 10.1177/09622802221111376

**Published:** 2022-07-01

**Authors:** Wei Liu, Frank Bretz, Mario Cortina-Borja

**Affiliations:** 1 Mathematical Sciences & Southampton Statistical Sciences Research Institute, University of Southampton, Southampton, UK; 2 Novartis Pharma AG, Basel, Switzerland; 3 Population, Policy and Practice Research and Teaching Department, Great Ormond Street Institute of Child Health, University College London, London, UK

We welcome the critique from Dr Wellek and Dr Jennen-Steinmetz. In particular, we share their concerns that a reference range (RR) should not be unnecessarily wide, leading to an avoidable low sensitivity of the RR. When a P-content and γ-confidence tolerance range (TR) is used as RR, this concern can be addressed by simply increasing the sample size n to improve its sensitivity since ‘as the sample size increases, the computed tolerance interval will approach the probability interval that actually contains the specified population proportion’.^1^ ${}^{\left(p.150\right)}$ To be more precise, it has been proposed^2^ to choose the sample size n to control the sensitivity of a (P,γ) TR in terms of

$$\mathrm{Pr}\{\;\mathrm{TR}\;\mathrm{contains}\;\mathrm{at}\;\mathrm{least}\;100P^{*}\text{\%}\;\mathrm{of}\;\mathrm{the}\;\mathrm{population}\;\}\leq \delta_{0}$$

where $P<P^{*}<1$ is pre-specified for a sufficiently small value of $\delta_{0}>0$. This sample size formula guarantees that there is no more than a small chance $\delta_{0}$ that the TR includes a proportion of $100P^{*}\text{\%}$ or more of the population. Tabulation of the sample sizes for the normal distribution can be found in Faulkenberry and Daly^3^ and Hahn and Meeker^1^ ${}^{\left(\mathrm{Ch}.9\right)}$ or easily computed by using the R package tolerance.^4^
